# Evidence of adaptation of maternofetal transport of glutamine relative to placental size in normal mice, and in those with fetal growth restriction

**DOI:** 10.1113/JP278226

**Published:** 2019-08-27

**Authors:** Kirsty R. McIntyre, Christina E. Hayward, Colin P. Sibley, Susan L. Greenwood, Mark R. Dilworth

**Affiliations:** ^1^ Maternal and Fetal Health Research Centre, Division of Developmental Biology and Medicine, School of Medical Sciences, Faculty of Biology, Medicine and Health The University of Manchester Manchester UK; ^2^ Manchester Academic Health Science Centre, St. Mary's Hospital Manchester University NHS Foundation Trust Manchester UK; ^3^ School of Medicine, Dentistry and Nursing, College of Medical, Veterinary and Life Sciences University of Glasgow Glasgow UK

**Keywords:** placenta, glutamine, glutamate, FGR, IUGR, mouse

## Abstract

**Key points:**

- Fetal growth restriction (FGR) is a major risk factor for stillbirth and has significant impact upon lifelong health.
- A small, poorly functioning placenta, as evidenced by reduced transport of nutrients to the baby, underpins FGR. It remains unclear how a small but normal placenta differs from the small FGR placenta in terms of ability to transfer nutrients to the fetus.
- Placental transport of glutamine and glutamate, key amino acids for fetal growth, was assessed in normal mice and those with FGR.
- Glutamine and glutamate transport was greater in the lightest *versus* heaviest placenta in a litter of normally grown mice. Placentas of mice with FGR had increased transport capacity in mid‐pregnancy, but this adaptation was insufficient in late pregnancy.
- Placental adaptations, in terms of increased nutrient transport (per gram) to compensate for small size, appear to achieve appropriate fetal growth in normal pregnancy. Failure of this adaptation might contribute to FGR.

**Abstract:**

Fetal growth restriction (FGR), a major risk factor for stillbirth, and neonatal and adulthood morbidity, is associated with reduced placental size and decreased placental nutrient transport. In mice, a small, normal placenta increases its nutrient transport, thus compensating for its reduced size and maintaining normal fetal growth. Whether this adaptation occurs for glutamine and glutamate, two key amino acids for placental metabolism and fetal growth, is unknown. Additionally, an assessment of placental transport of glutamine and glutamate between FGR and normal pregnancy is currently lacking. We thus tested the hypothesis that the transport of glutamine and glutamate would be increased (per gram of tissue) in a small normal placenta [C57BL6/J (wild‐type, WT) mice], but that this adaptation fails in the small dysfunctional placenta in FGR [insulin‐like growth factor 2 knockout (P0) mouse model of FGR]. In WT mice, comparing the lightest *versus* heaviest placenta in a litter, unidirectional maternofetal clearance (*K*_mf_) of ^14^C‐glutamine and ^14^C‐glutamate (^glutamine^ *K*_mf_ and ^glutamate^ *K*_mf_) was significantly higher at embryonic day (E) 18.5, in line with increased expression of LAT1, a glutamine transporter protein. In P0 mice, ^glutamine^ *K*_mf_ and ^glutamate^ *K*_mf_ were higher (P0 *versus* wild‐type littermates, WTL) at E15.5. At E18.5, ^glutamine^ *K*_mf_ remained elevated whereas ^glutamate^ *K*_mf_ was similar between groups. In summary, we provide evidence that ^glutamine^ *K*_mf_ and ^glutamate^ *K*_mf_ adapt according to placental size in WT mice. The placenta of the growth‐restricted P0 fetus also elevates transport capacity to compensate for size at E15.5, but this adaptation is insufficient at E18.5; this may contribute to decreased fetal growth.

## Key points

- Fetal growth restriction (FGR) is a major risk factor for stillbirth and has significant impact upon lifelong health.
- A small, poorly functioning placenta, as evidenced by reduced transport of nutrients to the baby, underpins FGR. It remains unclear how a small but normal placenta differs from the small FGR placenta in terms of ability to transfer nutrients to the fetus.
- Placental transport of glutamine and glutamate, key amino acids for fetal growth, was assessed in normal mice and those with FGR.
- Glutamine and glutamate transport was greater in the lightest *versus* heaviest placenta in a litter of normally grown mice. Placentas of mice with FGR had increased transport capacity in mid‐pregnancy, but this adaptation was insufficient in late pregnancy.
- Placental adaptations, in terms of increased nutrient transport (per gram) to compensate for small size, appear to achieve appropriate fetal growth in normal pregnancy. Failure of this adaptation might contribute to FGR.

## Introduction

Placental dysfunction is a major cause of pregnancy complications such as fetal growth restriction (FGR) (Mifsud & Sebire, 2014), which is characterised by poor growth *in utero* and has immediate and life‐long consequences (Veen *et al*. 1991; Barker, 2004; Thornton *et al*. 2004; Gardosi *et al*. 2013). One facet of this placental dysfunction is abnormal nutrient transport by the syncytiotrophoblast, the transporting epithelium of the placenta. This has been evidenced by studies in women demonstrating that the activity of key amino acid transport systems A and L is reduced in FGR (Glazier *et al*. 1997; Jansson *et al*. 1998). In addition, animal studies have shown that reductions in placental system A activity precede FGR (Jansson *et al*. 2006; Pantham *et al*. 2016) and that blockade of system A by substrate inhibition results in reduced fetal growth (Cramer *et al*. 2002). Collectively, these studies illustrate the importance of placental amino acid transport for appropriate growth of the fetus and suggest that reductions in amino acid transport may result in FGR.

The amino acids glutamine and glutamate are vital for metabolic processes and fetal growth. Glutamine is a conditionally essential amino acid during pregnancy and studies in sheep have demonstrated that it is transported to the fetus at one of the highest rates of all amino acids (Battaglia, 2002; Parimi & Kalhan, 2007), in keeping with its importance for fetal growth. Many essential cell processes such as nucleotide synthesis, pH homeostasis and gluconeogenesis require glutamine (Pochini *et al*. 2014). Glutamate is a precursor of glutamine and other molecules such as GABA, glutathione and 2‐oxoglutarate. Glutamate is also a neurotransmitter (Olsen & Sonnewald, 2015) and fetal plasma levels must be carefully controlled during fetal brain development because high levels of glutamate are neurotoxic (Tian *et al*. 2012).

In humans, fetal provision of glutamine and glutamate is achieved by amino acid recycling between the placenta and fetal liver. Both amino acids are transported from maternal blood into the placenta. However, evidence in humans, sheep and pigs suggests that glutamate is not transported directly from maternal to fetal blood (Pitkin *et al*. 1979; Vaughn *et al*. 1995; Battaglia, 2002; Self *et al*. 2004; Day *et al*. 2013) but instead is converted to glutamine in the syncytiotrophoblast. Glutamine then effluxes to the fetus and is converted back to glutamate by the fetal liver. Any glutamate not utilised by the fetus is taken up by the placenta to complete the cycle (Moores *et al*. 1994; Vaughn *et al*. 1995). The conversion of glutamine to/from glutamate within the placenta and fetal liver is important both for placental metabolism and for provision of amino acids to the fetus.

Placental transport of glutamine is mediated by several amino acid transport systems (system A, system N, system L, system y^+^L and system ASC) of overlapping specificity (Pochini *et al*. 2014). Systems A, N and L are known to contribute to placental uptake of glutamine from maternal blood, whereas the contribution of system y^+^L to glutamine transfer across the maternal‐facing microvillous membrane (MVM) of the human syncytiotrophoblast is negligible (Hill *et al*. 2014). Similarly, ASCT2, a transporter for system ASC, is predominantly localised to the basal membrane (BM; fetal‐facing) of the human syncytiotrophoblast (Johnson & Smith, 1988; Hoeltzli & Smith, 1989). Placental uptake of glutamate from maternal and fetal blood is mediated by system X_AG‐_. Recent studies in human placenta have also demonstrated a role for glutamate as a counter‐ion to mediate placental uptake of xenobiotics and hormones from fetal circulation (Lofthouse *et al*. 2015, 2018).

In normal human pregnancy, the concentration of most amino acids, including glutamine, is higher in umbilical venous blood (flowing from placenta to fetus) than in maternal plasma (Holm *et al*. 2017). There are conflicting reports regarding the concentration of glutamine in umbilical venous plasma in FGR compared with normal pregnancy. Ivorra *et al*. (2012) demonstrated a reduction in glutamine concentration in babies of low birth weight at term (below the 10th centile, corresponding to small for gestational age (SGA) infants compared to babies of normal birth weight. However, a recent study reported higher glutamine concentrations in venous cord blood of very low birth weight pre‐term babies compared with term infants of normal birth weight (Alexandre‐Gouabau *et al*. 2013); this finding was replicated by Sanz‐Cortés *et al*. (2013) in umbilical venous samples from both early‐ and late‐onset FGR, again defined by a birth weight < 10th centile. Both of these studies that demonstrated increased levels of glutamine in FGR were complicated by the inclusion of women diagnosed with pre‐eclampsia. Umbilical venous and arterial plasma glutamate (glutamic acid) concentration is reported to be reduced in human FGR (Alexandre‐Gouabau *et al*. 2013). In addition, Macnaught *et al*. (2015) used nuclear magnetic resonance imaging to show that the combined spectral contribution of glutamine/glutamate was reduced in placentas of human SGA infants *versus* normal birth weight and the authors proposed that this may be a biomarker of placental dysfunction *in utero*. A comprehensive assessment of placental transport of glutamine and glutamate *in vivo* in both normal pregnancy and FGR is currently lacking.

The pregnant mouse is a widely used model of human pregnancy. Like the human placenta, the mouse placenta is haemochorial (trophoblast tissue bathed in maternal blood) but in contrast to the human, the fetal portion of the mouse placenta is formed by two major but distinct zones: the junctional zone, primarily associated with endocrine function, and the labyrinthine zone, which is the major site of nutrient exchange (Enders & Blankenship, 1999; Malassine *et al*. 2003). Thus, the maternofetal nutrient exchange layer in the mouse, as its name suggests, has a labyrinthine‐like structure in contrast to the villous arrangement in the human placenta. The mouse placenta is classed as haemotrichorial, *versus* haemomonochorial in humans, because there are three trophoblast layers between maternal and fetal circulations: a discontinuous (trophoblast giant cell) layer I, syncytial layer II and fetal‐facing layer III. The apical membrane (layer II) is thought to be akin to the human syncytiotrophoblast MVM: both stain positively with alkaline phosphatase, and transporter proteins such as the GLUT1 glucose transporter have been localised to this layer (Enders, 1965; Takata & Hirano, 1997; Georgiades *et al*. 2002; Jansson *et al*. 2002). Layer III (basal membrane) is posited to be similar to the human syncytiotrophoblast BM, although this has not yet been confirmed due to a lack of a current method to isolate layer III in the mouse (for review see Dilworth & Sibley, 2013). Studies in pregnant mice, which enable assessment of nutrient delivery to the fetus using radioisotopes, have demonstrated that placental nutrient transport adapts according to placental size, thus maintaining appropriate fetal growth (Godfrey *et al*. 1998; Coan *et al*. 2008; Dilworth *et al*. 2010). Maternofetal transport of both calcium (Hayward *et al*. 2017) and the non‐metabolisable system A amino acid analogue methylaminoisobutyric acid (MeAIB) (Coan *et al*. 2008) is increased, per gram placenta, in the lightest *versus* heaviest placentas in a litter of wild‐type C57Bl/6J (WT) mice. This adaptation helps to ensure that the fetus is born within a normal birth weight range. As a substrate of system A, one would hypothesise that glutamine transport also adapts in relation to placental size in WT mice, but, prior to the current study, this has not been investigated. Furthermore, no studies, in mice or human, have explored whether the glutamate transporter system X_AG‐_ adapts according to placental size.

Improving our knowledge of the physiological processes that regulate nutrient provision to the fetus in normal pregnancy can inform our understanding of why amino acid transport is reduced in FGR. Recent research in mice suggests that the mechanisms underpinning placental adaptations, at least in terms of placental calcium transfer, are distinct in normally grown and growth restricted fetuses (Hayward *et al*. 2018). Therefore, an assessment of maternofetal placental glutamine and glutamate transfer in a model of FGR provides the opportunity to assess whether any adaptations present in normal mice are altered in placentas of growth‐restricted fetuses. Here we chose a genetic mouse model of FGR, the placental‐specific insulin‐like growth factor 2 knockout (P0) mouse, to examine maternofetal transfer of glutamine and glutamate. The P0 mouse is a model of late‐onset FGR, pups weigh 30% less than WT at birth, which gives rise to mixed litters of P0 (growth restricted) and wild‐type littermates (WTL). P0 fetuses have significantly lighter placentas prior to the onset of growth restriction (Constância *et al*. 2002). In this model maternofetal transfer of glucose and MeAIB is significantly higher (P0 *versus* WTL) in mid‐gestation, a time point when normal fetal growth is maintained. However, this functional adaptation is lost by term and FGR ensues (Constância *et al*. 2002, 2005).

The aims of this study were to assess maternofetal glutamine and glutamate transport in normal pregnancy (WT mice) and in a model of FGR (P0 mice). We tested the hypothesis that when comparing lightest *versus* heaviest placentas in a normal (WT) litter, the lightest placentas functionally adapt their glutamine and glutamate transport capacity to maintain appropriate fetal growth. We also hypothesised that in the P0 mouse (comparing P0 and WTL), this adaptation is disrupted or absent, which contributes to FGR. To investigate potential mechanisms, the abundance of putative glutamine and glutamate transporter proteins (systems N and L, and X_AG‐_, respectively) was assessed by Western blot.

## Materials and methods

### Animals

All experimental procedures were performed in accordance with the UK Animal (Scientific Procedures) Act of 1986 under Home Office project licences PPL 40/3385 and P9755892D and were approved by the local ethical review panel of the University of Manchester. The methods described herein adhere to the ARRIVE guidelines (Kilkenny *et al*. 2010).

Mice were communally housed (except for stud males that were individually housed) in individually ventilated cages in rooms with a 12 h light–dark cycle at 21–23°C. Mice were provided with nesting material and had free access to food (BK001 diet, Special Diet Services, Essex, UK) and water (Hydropac watering system, Lab products Inc., Seaford, Delaware, USA).

Wild‐type (WT) C57BL/6J (Envigo, Huntington, UK) female (10–16 weeks old) and male (10–26 weeks old) mice were mated, and the lightest *versus* heaviest placentas in a litter were compared to examine the extremes of placental weight in normal pregnancy. The first day of gestation was determined by the discovery of a copulation plug [embryonic day (E) 0.5; term = E19.5]. Pregnant female mice were killed at E15.5 or E18.5 (chosen as peak growth of the fetus occurs between these two time points) by cervical dislocation alone, or terminal anaesthesia followed by exsanguination (cardiac puncture) and cervical dislocation (only for unidirectional maternofetal clearance measures; see below) appropriate under ASPA schedule 1. A laparotomy and hysterotomy was then performed. All fetuses were rapidly killed by cervical dislocation. Placentas and fetuses were rapidly harvested, blotted and wet weights measured to identify the lightest and heaviest placentas in a litter. Fetal tail tips were collected from all fetuses and stored at −20°C for determination of sex. Placentas and fetuses were immediately snap frozen and stored at −80°C. Fetal weight histograms were constructed and a non‐linear regression was performed (Gaussian distribution) from which individualised fetal weight centiles were calculated as described previously (Dilworth *et al*. 2011).

Placental‐specific insulin‐like growth factor 2 (*Igf2*) (P0) knockout mice, which had deletion of the U2 exon of the *Igf2* gene, were generated as previously described (Constância *et al*. 2000) and were a kind gift from Dr Miguel Constância and Professor Wolf Reik, University of Cambridge. C57BL/6J female mice (8–16 weeks old) and males heterozygous for the P0 deletion (10–101 weeks old) were mated and produced mixed litters of growth‐restricted (P0) and wild‐type littermate (WTL) fetuses. Embryonic day was determined as above. At E15.5 or E18.5 pregnant female mice were killed and a laparotomy and hysterotomy was performed. Fetal tail tips were collected from all fetuses and stored at −20°C for genotype determination. Placentas and fetuses were weighed, snap frozen and stored at −80°C. The aim of the study, comparing lightest *versus* heaviest placentas or those from P0 *versus* WTL mice within a single litter, meant that randomisation or blinding of the samples was not possible.

### Determination of sex

The sex of each fetus was determined according to a previously published genotyping protocol (Kunieda *et al*. 1992); genomic DNA was extracted from fetal tail tips using DirectPCR lysis reagent (mouse tail) (Bioquote, York, UK) containing proteinase K (Qiagen, Manchester, UK) and MyTaq Red Mix (Bioline Ltd, London, UK) with primers specific to gene sequences for SRY (Y chromosome; 404 bp; SRY2 5′‐TCTTAAACTCTGAAGAAGAGAC‐3′, SRY4 5′‐GTCTTGCCTGTATGTGATGG‐3′) and NDS (X chromosome; 244 bp; NDS3 5′‐GAGTGCCTCATCTATACTTACAG‐3′, NDS4 5′‐TCTAGTTCATTGTTGATTAGTTGC‐3′) (Kunieda *et al*. 1992). PCR conditions were as follows; 4 min denaturation at 94°C; 35 cycles of 1 min at 94°C, 1 min at 55°C and 1 min at 72°C; and 10 min final extension at 72°C. Samples were run on a 2% agarose gel and bands were visualised using an InGenius transilluminator (Sygene Bio, Cambridge, UK).

### Genotyping of P0 knockout mice

Genotype (P0 or WTL) was determined for all fetuses from P0 dams according to a previously published genotyping protocol (Constância *et al*. 2005; Dilworth *et al*. 2010). In brief, genomic DNA was extracted from fetal tail tips using a DNeasy kit (Qiagen). *Igf2* P0^+/−^ mutants were identified with a specific primer pair to amplify a 740 bp fragment across the 5 kb deletion (P0 dF 5′‐TCCTGTACCTCCTAACTACCAC‐3′ and P0 dR 5′‐GAGCCAGAAGCAAACT‐3′) and a primer to amplify a 495 bp fragment from the WT allele (5′‐CAATCTGCTCCTGCCTG‐3′). PCR conditions were as follows: 4 min denaturation at 94°C; 35 cycles of 1 min at 94°C, 1 min at 56°C and 1 min at 72°C; and 10 min final extension at 72°C. Samples were loaded with bromophenol blue and run on a 1.5% agarose gel. Bands were visualised using an InGenius transilluminator (Sygene Bio).

### Unidirectional maternofetal clearance of ^14^C‐glutamine and ^14^C‐glutamate

Unidirectional maternofetal clearance of ^14^C‐glutamine and ^14^C‐glutamate across the intact placenta was evaluated at E15.5 and E18.5 as described previously (Flexner & Pohl, 1941; Bond *et al*. 2008; Dilworth *et al*. 2010). Mice were anaesthetised with a 300 µl intraperitoneal injection of 1:1:2 combination of fentanyl/fluanisone (Hypnorm, VetPharma Ltd, Leeds, UK), Midazolam (Roche, UK) and sterile H_2_O (Braun medical Inc., Pennsylvania, USA). Then, 0.023 mBq ^14^C‐glutamine and 0.046 mBq ^14^C‐glutamate was made up in 100 µl sterile phosphate‐buffered saline (PBS) and administered via tail vein cannula confirmed by pre‐ and post‐weighing of the syringe (Kern EW 150‐3M precision balance, Kern and Sohn, Germany). At approximately 2 min after injection (between 90 s and 4 min for ^14^C‐glutamine; 90 s and 3 min for ^14^C‐glutamate), dams were exsanguinated by cardiac puncture; the maternal blood sample was obtained and centrifuged at 5000 rpm (1845 *g*) for 5 min to obtain plasma. All fetuses and placentas were collected, assessed for total radiolabel accumulation and compared to a maternal plasma disappearance curve (see below). Unidirectional maternofetal clearance (^glutamate^ *K*_mf_ or ^glutamate^ *K*_mf_; µL/min/g placenta) was calculated as previously described (Dilworth *et al*. 2010). Unidirectional maternofetal clearance (*K*_mf_) is the volume of maternal plasma theoretically cleared of isotope per unit time, expressed relative to individual placental weight (per gram of placenta). It can also be helpful to express maternofetal transfer relative to fetal weight (µL/min/g fetus) or irrespective of fetal or placental weight (total radioisotope transfer) to investigate the relationship between nutrient transfer and fetal/placental weight. The total amount of nutrients that reach the fetus is a key determinant of fetal growth. All clearance experiments were carried out between the hours of 08:00 and 12:00 in the same theatre room. Comparisons were made between: the lightest and heaviest placentas from each litter at E15.5 (glutamine *N* = 9 litters, *n* = 9 lightest *versus n* = 9 heaviest; glutamate *N* = 6 litters, *n* = 6 lightest *versus n* = 6 heaviest) and at E18.5 (glutamine *N* = 8 litters, *n* = 8 lightest *versus n* = 8 heaviest; glutamate *N* = 7 litters, *n* = 7 lightest *versus n* = 7 heaviest); and between litter averages for P0 and WTL at E15.5 (glutamine *N* = 6 litters; *n* = 25 P0, *n* = 25 WTL; glutamate *N* = 7 litters; *n* = 25 P0, *n* = 28 WTL) and at E18.5 (glutamine *N* = 6 litters; *n* = 26 P0, *n* = 19 WTL; glutamate *N* = 7 litters; *n* = 33 P0, *n* = 24 WTL). Only those litters with at least two P0 and two WTL were included for analyses.

### Maternal plasma ^14^C‐glutamine and ^14^C‐glutamate disappearance curve

The time at which clearance of ^14^C‐glutamine and ^14^C‐glutamate from the maternal circulation was linear was determined by the construction of a ^14^C maternal plasma disappearance curve at E15.5 and E18.5 (glutamine *N* = 27 WT, *N* = 19 P0; glutamate *N* = 23 WT, *N* = 17 P0) and fitted to a one‐phase exponential decay model (*r* ^2^ > 0.5), as described previously (Bond *et al*. 2006). There was no difference in the isotope disappearance curves in WT and P0 mice (data not shown).

### Protein expression

The lightest and heaviest placentas in a WT litter (E15.5 *N* = 7 litters, E18.5 *N* = 8 litters; paired comparison between lightest and heaviest placentas in each litter), and the placentas of P0 and WTL mice (E15.5 *N* = 8 litters, E18.5 *N* = 8 litters; one paired P0 and WTL placenta per litter selected at random) were homogenised and processed as described previously (Bond *et al*. 2006). Briefly, whole homogenates were separated, by means of centrifugation, to obtain a membrane‐enriched fraction. SDS‐PAGE was performed followed by electrotransfer to Immobilon‐FL PVDF membranes (Millipore UK Ltd, Watford, UK). Primary antibodies included: rabbit polyclonal LAT1 (0.5 µg/ml; KE026; TransGenic Inc, Tokyo, Japan); rabbit polyclonal LAT2 (2 µg/ml; abx121147; Abbexa, Cambridge, UK for WT mice, due to discontinuation, 2 µg/ml; ab75610; Abcam, Cambridge, UK was used for P0 studies); SNAT5 (1.4–1.8 µg/ml; ab72717; Abcam); EAAT1 (1 µg/ml; ab416; Abcam); and rabbit monoclonal EAAT2 (2.69 µg/ml; ab178401; Abcam). Rabbit polyclonal β‐actin (2 µg/ml; ab8227; Abcam) or β‐tubulin (2 µg/ml; ab6046; Abcam) was used as a loading control; when used no difference was observed in β‐actin or β‐tubulin expression between experimental groups. Negative controls were constructed by omission of primary antibody during optimisation. Each experimental run included a positive (mouse brain whole homogenate or human MVM isolate) and negative (distilled H_2_O) control. Immunoreactive species were detected with fluorescence‐conjugated secondary antibodies (Li‐COR Biosciences, Cambridge, UK) and membranes were imaged using an Odyssey Sa Infrared Imaging System (Li‐COR). Signal density was measured using Image Studio Lite (Li‐COR). All signals were in the linear range of detection. Comparisons were made separately between lightest and heaviest (E15.5 *N* = 6/7 litters, E18.5 *N* = 4/8 litters), and P0 and WTL (E15.5 *N* = 8 litters, E18.5 *N* = 6/8 litters).

### Statistical analysis

Data from WT litters are presented as the lightest placenta as a percentage of the heaviest in a litter (dotted line is heaviest = 100%). Data from P0 litters are presented as the litter average of P0 fetuses as a percentage of WTL (dotted line corresponds to WTL = 100%). Data are shown as median (range) where the experimental *N* = number of litters and *n* = number of placentas or fetuses. Litter averages were used for comparisons between P0 and WTL, and thus inclusion criteria were a minimum of two P0 and two WTL per litter. The solid line on graphs represents the median value. To calculate sample sizes (80% statistical power at a 5% significance level) per experiment, a standardised difference was calculated based upon data from our previously published studies (Dilworth *et al*. 2010; Hayward *et al*. 2017). Data were analysed by Wilcoxon signed‐rank test *versus* 100% or Mann–Whitney test. *P < *0.05 was considered statistically significant.

## Results

### Placental and fetal weights in the lightest *versus* heaviest placentas in a WT litter

**Figure 1:**
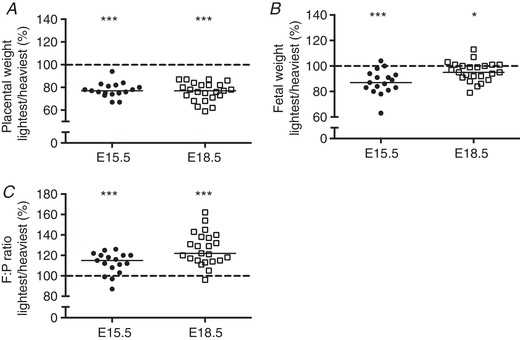
Placental weight (*A*), fetal weight (*B*) and fetal weight:placental weight (F:P) ratio (*C*) in the lightest and heaviest placentas in a WT litter at embryonic day (E)15.5 and E18.5 Data are presented as the lightest placenta as a percentage of the heaviest in a litter. Black line = median; dotted line, 100% = heaviest placenta. ^*^ *P < *0.05, ^***^ *P < *0.001 Wilcoxon signed‐rank test *versus* 100%.

The lightest placenta in a WT litter weighed on average 23% less than the heaviest at both E15.5 (*N* = 17) and E18.5 (*N* = 23) (Table 1; Fig. 1 *A*) (*P < *0.001). Fetuses from the lightest placentas were significantly lighter at E15.5 and E18.5, yet the magnitude of this difference was smaller towards term (13%, *P < *0.001 at E15.5; 5%, *P < *0.05 at E18.5) (Table 1; Fig. 1 *B*). Fetuses with the lightest placentas had lower fetal weight centiles compared with heaviest placentas in a litter at E15.5 (median 38.4 *versus* 58.2; *P* < 0.001) and E18.5 (median 51.2 *versus* 71.0; *P* < 0.01) but were still within the normal range (10–90th centile) (data not shown). At both gestational ages, the fetal weight:placental weight (F:P) ratio, a proxy of placental efficiency, was significantly higher for fetuses with the lightest placentas (*P < *0.001) (Table 1; Fig. 1 *C*). There was no difference in litter size between E15.5 (median = 7 fetuses, range 4–9) and E18.5 (median = 7 fetuses, range 6–10).

**Table 1:** Placental weight, fetal weight and fetal weight:placental weight (F:P) ratio in the lightest and heaviest placentas in a WT litter at embryonic day (E)15.5 (*N* = 17) and E18.5 (*N* = 23)

	E15.5			E18.5		
	Lightest	Heaviest	*P* value	Lightest	Heaviest	*P* value
Placental weight (g)	0.078	0.102	<0.001	0.070	0.090	<0.001
	(0.070–0.091)	(0.080–0.135)		(0.052–0.080)	(0.079–0.117)	
Fetal weight (g)	0.387	0.422	<0.001	1.184	1.238	0.014
	(0.245–0.563)	(0.343–0.602)		(0.959–1.355)	(1.001–1.384)	
F:P ratio	4.8	4.0	<0.001	17.0	13.7	<0.001
	(3.5–6.5)	(3.0–5.8)		(12.8–21.6)	(10.5–15.4)	

Data are median (range). Data were analysed by Wilcoxon signed‐rank test *versus* 100%.

### Unidirectional maternofetal glutamine and glutamate clearance across the lightest and heaviest placentas in a WT litter

**Figure 2:**
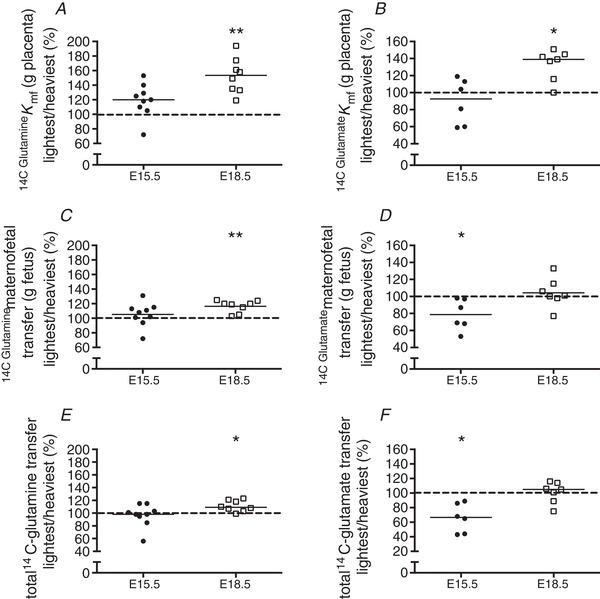
Unidirectional maternofetal clearance (*K*_mf_)_,_ maternofetal transfer and total transfer of glutamine (*A*, *C*, *E*) and glutamate (*B*, *D*, *F*) in the lightest and heaviest placentas in a WT litter at embryonic days (E)15.5 and E18.5 Data are expressed as the lightest placenta in a litter as a percentage of the heaviest placenta in a litter. Black line = median; dotted line, 100% = heaviest placenta. ^*^ *P* < 0.05, ^**^ *P* < 0.01 Wilcoxon signed‐rank test *versus* 100%.

Unidirectional maternofetal clearance (*K*_mf_) of glutamine per gram of placenta (µL/min/g placenta) was significantly higher (*P *< 0.01) in lightest *versus* heaviest placentas at E18.5, but was not different at E15.5 (Fig. 2 *A*). Maternofetal transfer of glutamine, both per gram of fetus (µL/min/g fetus) (Fig. 2 *C*) and total transfer (µL/min) (Fig. 2 *E*), was significantly higher in the lightest compared with the heaviest placentas at E18.5 (*P* < 0.01 and *P* < 0.05, respectively).

Unidirectional maternofetal clearance (*K*_mf_) of glutamate per gram of placenta was significantly higher in the lightest compared with the heaviest placentas at E18.5 (*P < *0.05) but not at E15.5 (Fig. 2 *B*). Maternofetal transfer of glutamate per gram of fetus (µL/min/g fetus) (Fig. 2 *D*) and total maternofetal transfer of glutamate (µL/min) (Fig. 2 *F*) were significantly lower in the lightest placenta at E15.5 (*P* < 0.05), but not significantly different at E18.5.

The lightest placentas in a litter are more frequently from female fetuses, and similarly a greater proportion of the heaviest placentas in a litter belong to male fetuses. In this study, 69% (9/13) and 72% (13/18) of the lightest placentas in a litter were from female fetuses at E15.5 and E18.5, respectively. At E15.5, 69% (9/13) of the heaviest placentas were from male fetuses, this figure was 83% (15/18) at E18.5. However, unidirectional maternofetal clearance (*K*_mf_, µL/min/g placenta) was not different according to sex at either E15.5 or E18.5 (litter mean (range) of female fetuses as a percentage of male fetuses (100%), with a minimum of two of each sex per litter: ^glutamine^ *K*_mf_ E15.5 107% (86–120%), E18.5 119% (89–141%); and ^glutamate^ *K*_mf_ E15.5 105% (87–123%), E18.5 111% (91–136%) (*P > *0.05 in all instances).

### Expression of placental glutamine and glutamate transporter proteins in the lightest *versus* heaviest placentas in a WT litter

**Figure 3:**
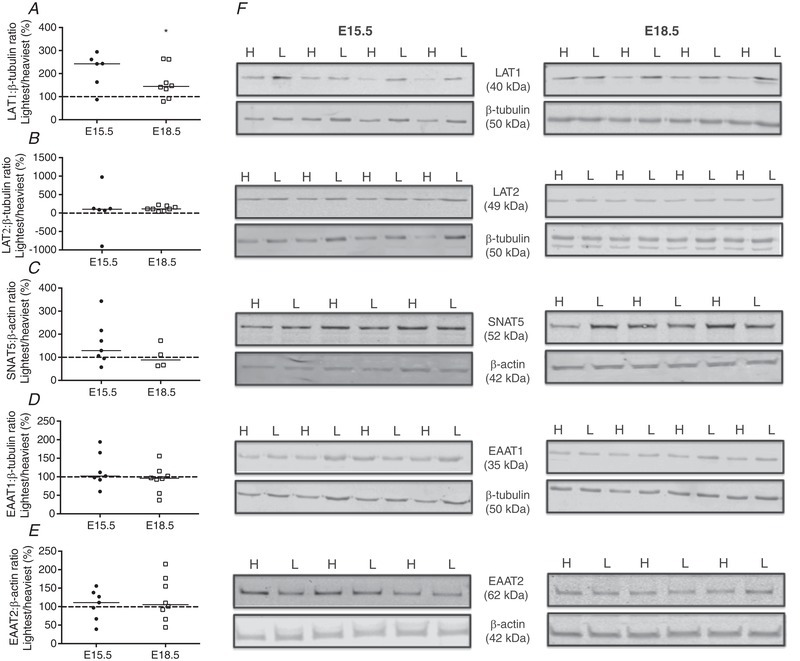
Expression of glutamine [LAT1 (*A*), LAT2 (*B*), SNAT5 (*C*)] and glutamate [EAAT1 (*D*), EAAT2 (*E*)] transporter proteins in membrane‐enriched placental homogenates (expressed as percentage of lightest (L) *versus* heaviest (H) placentas in a WT litter) at embryonic day (E)15.5 (*N* = 6/7) and E18.5 (*N* = 4/8) Densitometric analysis is expressed as a ratio of β‐tubulin or β‐actin signal. *F*, representative Western blots of LAT1, LAT2, SNAT5, EAAT1 and EAAT2 at E15.5 and E18.5 with the corresponding housekeeper protein. Black line = median; dotted line 100% = heaviest placenta. ^*^ *P < *0.05; Wilcoxon signed‐rank test *versus* 100%.

Western blotting was used to determine whether functional changes were due to altered transporter protein abundance. Paired comparisons were made between the lightest and heaviest placentas in a WT litter (see Fig. 3 *F* for representative blots). There was no difference in the expression of the glutamine transporters LAT1, LAT2 and SNAT5 (Fig. 3 *A–C*), and the glutamate transporters EAAT1 and EAAT2 (Fig. 3 *D*, *E*) at E15.5. Expression of LAT2, SNAT5, EAAT1 and EAAT2 was also no different at E18.5. However, there was a statistically significant increase in LAT1 protein expression at E18.5 in the lightest *versus* heaviest placentas (Fig. 3 *A*).

### Placental and fetal weights in P0 *versus* WTL

**Figure 4:**
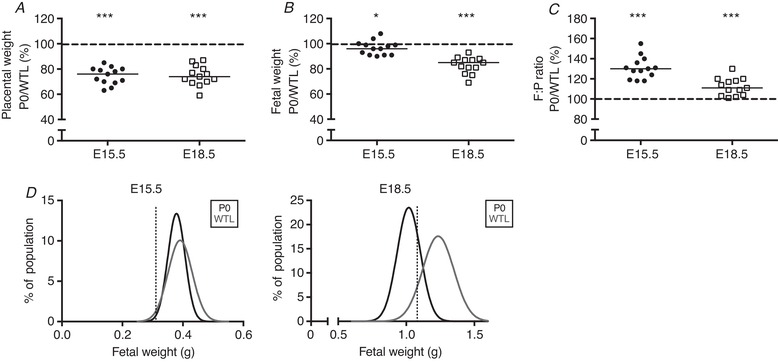
Placental weight (*A*), fetal weight (*B*) and fetal weight:placental weight (F:P) ratio (*C*) in P0 and wild‐type littermates (WTL) at embryonic day (E)15.5 and E18.5 Data are presented as the litter average of P0 as a percentage of the litter average of WTL. Black line = median; dotted line, 100% = WTL. ^*^ *P < *0.05, ^***^ *P < *0.001; Wilcoxon signed‐rank test *versus* 100%. *D*, fetal weight distribution curves for P0 and WTL fetuses are shown for E15.5 and E18.5. The dotted line represents the 5th centile of WTL fetal weights, which was 0.33 g at E15.5 and 1.04 g at E18.5.

Placentas from P0 fetuses were significantly lighter (*P < *0.001) at both gestational ages, weighing 76% of those from WTL at E15.5, and 74% towards term (E18.5) (Table 2; Fig. 4 *A*). At E15.5 P0 fetuses weighed significantly less (96%; *P* < 0.05) than WTL; this difference was more pronounced towards term (E18.5 = 85%; *P* < 0.001) (Table 2; Fig. 4 *B*). P0 fetuses had a significantly higher F:P ratio compared with WTL at E15.5 and E18.5 (*P* < 0.001) (Table 2; Fig. 4 *C*). Fetal weight histograms constructed using non‐linear regression (Gaussian distribution) revealed that at E15.5 8% of P0 fetuses were below the 5th centile of WTL weights (0.33 g), a clinically relevant threshold for FGR. By E18.5 the majority of P0 fetuses (57%) were below the 5th centile of WTL weights (1.04 g) (Fig. 4 *D*). There was no difference in litter size between gestational ages (E15.5 median = 8 fetuses, range 6–10; E18.5 median = 8 fetuses, range 4–12).

**Table 2:** Placental weight, fetal weight and fetal weight:placental weight (F:P) ratio in P0 and wild‐type littermates (WTL) at embryonic day (E)15.5 (*N* = 13) and E18.5 (*N* = 13)

	E15.5			E18.5		
	P0	WTL	*P* value	P0	WTL	*P* value
Placental weight (g)	0.077	0.104	<0.001	0.066	0.094	<0.001
	(0.065–0.086)	(0.089–0.112)		(0.057–0.078)	(0.076–0.104)	
Fetal weight (g)	0.363	0.387	0.02	1.031	1.220	<0.001
	(0.351–0.426)	(0.346–0.432)		(0.837–1.091)	(1.108–1.381)	
F:P ratio	4.9	3.9	<0.001	15.2	13.6	<0.001
	(4.5–6.2)	(3.3–4.6)		(13.4–16.9)	(10.8–15.3)	

Data are median (range). Data were analysed by Wilcoxon signed‐rank test *versus* 100%.

### Unidirectional maternofetal glutamine and glutamate clearance in P0 *versus* WTL

**Figure 5:**
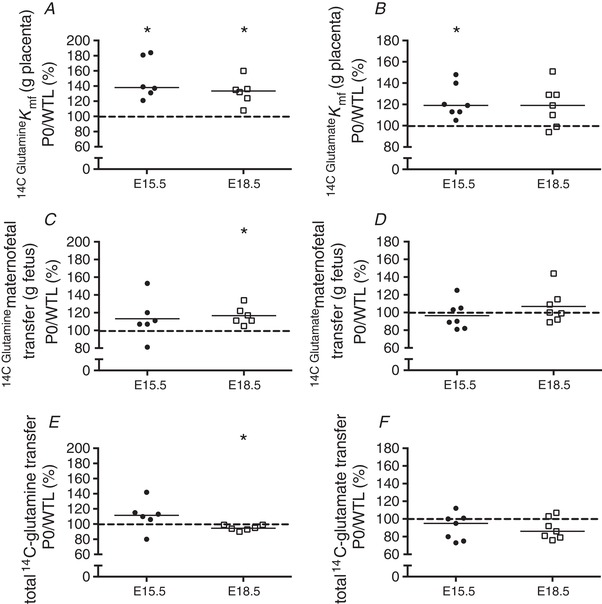
Unidirectional maternofetal clearance (*K*_mf_)_,_ maternofetal transfer and total transfer of glutamine (*A*, *C*, *E*) and glutamate (*B*, *D*, *F*) in P0 and wild‐type littermates (WTL) at embryonic days (E)15.5 and E18.5 Data are expressed as the litter mean of placentas from P0 fetuses as percentage of the litter mean of WTL. Black line = median dotted line, 100% = litter mean of WTL placentas. ^*^ *P < *0.05 Wilcoxon signed‐rank test *versus* 100%.

Unidirectional maternofetal clearance (*K*_mf_; µl/min/per g placenta) of glutamine was significantly higher for P0 compared with WTL fetuses at E15.5 and E18.5 (*P < *0.05) (Fig. 5 *A*). Maternofetal glutamine transfer per gram of fetus (µL/min/g fetus) (Fig. 5 *C*) was significantly higher (*P < *0.05; P0 *versus* WTL) at E18.5 only whereas total maternofetal transfer of glutamine (µL/min) (Fig. 5 *E*) was significantly lower at E18.5 (*P < *0.05). Unidirectional maternofetal clearance (*K*_mf_) of glutamate was significantly higher for P0 compared with WTL (*P < *0.05) at E15.5 but not at E18.5 (Fig. 5 *B*). There was no difference in either maternofetal transfer of glutamate per gram of fetus (µL/min/g fetus) (Fig. 5 *D*) or total maternofetal transfer of glutamate (µL/min) (Fig. 5 *F*) between P0 and WTL at E15.5 or E18.5.

### Expression of placental glutamine and glutamate transporter proteins in P0 *versus* WTL

**Figure 6:**
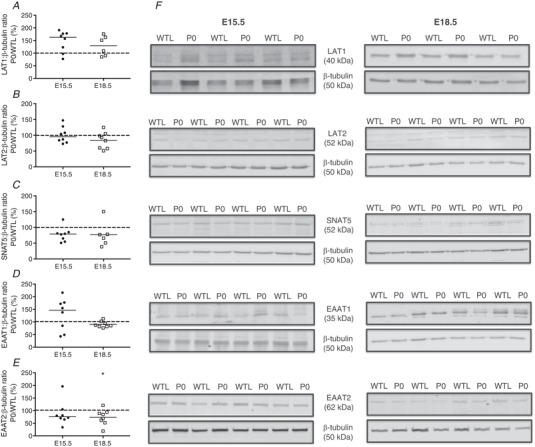
Expression of glutamine [LAT1 (*A*), LAT2 (*B*), SNAT5 (*C*)] and glutamate [EAAT1 (*D*), EAAT2 (*E*)] transporter proteins in membrane‐enriched placental homogenates [expressed as percentage of P0 *versus* wild‐type littermates (WTL)] at embryonic day (E)15.5 (*N* = 8) and E18.5 (*N* = 6/8) Densitometric analysis is expressed as a ratio of β‐tubulin signal. *F*, representative Western blots of LAT1, LAT2, SNAT5, EAAT1 and EAAT2 at E15.5 and E18.5 with the corresponding housekeeper protein. Black line = median; dotted line 100% = WTL placenta. ^*^ *P < *0.05; Wilcoxon signed‐rank test *versus* 100%.

One P0 and one WTL placenta per litter was selected at random for protein expression studies (see Fig. 6 *F* for representative blots). The expression of glutamine transporter proteins (LAT1, LAT2, SNAT5) in placental membrane preparations was no different between P0 and WTL at either gestational age (E15.5 or E18.5) (Fig. 6 *A–C*). There was also no difference in expression of glutamate transporter protein EAAT1 at E15.5 or E18.5 (Fig. 6 *D*). EAAT2 expression was significantly lower at E18.5 (Fig. 6 *E*).

## Discussion

Glutamine and glutamate are essential for the cellular and metabolic requirements of the placenta and fetus (Parimi & Kalhan, 2007). Perturbations in placental transport of these amino acids may contribute to reduced fetal growth (FGR). Maternofetal transfer of glutamine and glutamate was evaluated by comparing the extremes of placental weight in WT mice, and P0 *versus* WTL in the P0 mouse model of FGR. In WT mice, the lightest placentas had a greater unidirectional maternofetal clearance of glutamine and glutamate, per gram placenta, relative to the heaviest placentas within the same litter. This adaptation was sufficient to ensure appropriate delivery of glutamine and glutamate to the fetus at E18.5. For glutamate, this represents a normalisation of maternofetal transfer following an observed reduction at E15.5. In the P0 knockout mouse model of FGR, increased maternofetal clearance of glutamine, per gram placenta, was apparent at both E15.5 and E18.5 in P0 *versus* WTL but this adaptation was unable to fully compensate for the reduced placental size at E18.5, as evidenced by reduced total maternofetal transfer of glutamine at E18.5.

These findings are consistent with existing evidence that the small, normal mouse placenta can functionally adapt to meet fetal nutrient requirements. This adaptation results in a fetus that, whilst still significantly lighter than a fetus from the largest placenta in a litter (by 6%), remains within a normal birth weight range at term, i.e. is not growth‐restricted as is the case for P0 fetuses. A dominant role of LAT1, expression of which was higher in the lightest *versus* heaviest placentas towards term, is to maintain the amino acid pool within the cell. LAT1 is involved in the uptake of large neutral amino acids and works in concert with Na^+^‐dependent unidirectional transporters such as systems A and N (Verrey, 2003; Pochini *et al*. 2014). In the context of the study results, LAT1 may play a role in maintaining the amino acid pool within the syncytiotrophoblast of the lightest placentas, thus contributing to the amount of amino acids available for exchange between the placenta and fetus, whilst net uptake is achieved via other transporter mechanisms. A role for system A transporter isoforms cannot be ruled out; a lack of commercially available antibodies that reliably produce bands at the predicted size for SNAT1, SNAT2 and SNAT4 means that we were unable to quantify expression of these transporters. Previous assessment of gene expression in the lightest *versus* heaviest placentas in a WT litter found that expression of the gene encoding SNAT2 (slc38a2) is higher in the lightest *versus* heaviest placentas in a WT litter (other isoforms expressed in the placenta, slc38a1/SNAT1 and slc38a4/SNAT4, were unchanged) (Coan *et al*. 2008). SNAT1 and SNAT2 have broad substrate specificity, including glutamine, whilst SNAT4 is generally not considered to transport glutamine (Schiöth *et al*. 2013).

It is known that homeostatic control of amino acid concentrations is maintained by sophisticated regulatory pathways such as the mechanistic target of rapamycin (mTOR). mTOR is part of a nutrient sensing pathway stimulated by nutrients such as amino acids and growth factors. Inhibition of mTOR, which mimics the reduced activity observed in FGR (Roos *et al*. 2007), leads to decreased system A, L and β transporter activity but no difference in transporter expression (Roos *et al*. 2009), which suggests that mTOR regulates amino acid transporters at the post‐translational stage. Moreover, the downstream effects of activated mTOR complex 1 (mTORC1) include inhibition of the NEDD4‐2 complex leading to reduced ubiquitination and degradation of amino acid transporters such as LAT1 and SNAT2 by the proteasome. In light of the findings in WT mice, i.e. altered *K*_mf_ of glutamine and glutamate in the absence of differences in overall protein transporter abundance, aside from raised LAT1 expression, the mTOR signalling pathway is a candidate mechanism by which glutamine clearance is modified and we are currently investigating whether altered mTOR activity is responsible for the changes in *K*_mf_ of glutamine and glutamate.

In the current study we were unable to account for the higher maternofetal glutamate clearance in the lightest WT placentas towards term; expression of transporters important for glutamate transport were no different at either gestational age studied. In the CNS, EAAT2 is post‐translationally modified by the addition of a small ubiquitin‐like modifier (SUMO) to an accessible lysine. SUMOylation causes the transporter to be internalized and available for response from an intracellular pool (Foran *et al*. 2014). There is also evidence that system X_AG‐_ activity is increased during periods of amino acid deprivation in a kidney cell line (Plakidou‐Dymock & McGivan, 1993). It is reasonable to suggest that post‐translational modifications and/or signalling pathways are important in mediating functional changes in glutamate delivery in the lightest placentas.

Considering the results from the current study, it is conceivable that there are distinct mechanisms driving glutamine and glutamate provision to the fetus. The total amount of glutamine transfer to the fetus (irrespective of fetal or placental weight) was significantly higher for the lightest compared with the heaviest placentas at E18.5, which highlights the importance of this amino acid for fetal growth. Conversely, glutamate transfer was normalised towards term following a significant decrease earlier in gestation.

Consistent with previously published literature in this area (Hayward *et al*. 2017), the lightest placentas in a litter were more often from females, and the heaviest placentas predominantly from males. An assessment of the contribution of sex, by comparing maternofetal clearance (*K*_mf_) for the litter mean of females relative to the litter mean of males, did not yield any significant differences between groups. Therefore, it is reasonable to conclude that the contribution of placental size is greater than that of the sex of the fetus in relation to the functional adaptations reported here, an observation that we have previously seen for maternofetal transport of calcium (Hayward *et al*. 2017). Within the constraints of a relatively small WT litter (average of seven fetuses per litter) there is somewhat limited variation between the lightest female placenta and the heaviest female placenta, and likewise for males. Practically it was not feasible to compare these groups in the current study; a study of this kind would thus probably require greater numbers to detect differences and a strength of the current study is the ability to make paired observations within a litter.

We hypothesised that any functional adaptations present in the P0 mouse at E15.5 would be lost towards term, in alignment with apparent growth restriction at this time. The data in terms of maternofetal clearance of glutamate, per gram placenta, support this concept: ^glutamate^ *K*_mf_ was significantly higher (P0 *versus* WTL) at E15.5 but similar between groups at E18.5. Contrary to this hypothesis, unidirectional maternofetal clearance of glutamine (^glutamine^ *K*_mf_), per gram placenta, was significantly higher in P0 *versus* WTL both at E15.5 and at E18.5. However, at E18.5, this adaptation was not enough to normalise total maternofetal transfer of glutamine in P0, which was reduced *versus* WTL. These placental adaptations, in terms of nutrient transport in P0 mice, were ultimately insufficient to overcome the challenge of a small, pathological placenta; at E18.5 P0 fetuses were significantly smaller than WTL.

Previous studies have shown that relative to placental size, activity of system A (MeAIB clearance) is comparable between P0 and WTL at E18.5 (Constância *et al*. 2005). It is therefore plausible that system L or N are the main drivers of the altered glutamine transfer at this time point. However, our investigation of potential mechanisms did not support this – we did not find any significant differences in system L or N protein expression at either gestational age. This suggests a role of other mechanisms, such as the post‐translational modifications described above. Recent evidence focused upon maternofetal transport of calcium indicates that the underlying mechanisms behind these adaptations in WT mice are distinct from those in P0 mice (Hayward *et al*. 2018). The data presented here indicate that no significant differences in LAT1 expression were observed between the placentas of P0 and WTL, in contrast to lightest *versus* heaviest placentas. Furthermore, expression of the glutamate transporter protein EAAT2 was significantly lower at E18.5 in P0 compared with WTL, whereas no difference was observed in WT litters. In the P0 mouse close to term there is higher glutamine but not glutamate clearance, per gram placenta, indicating that the increase is selective for certain amino acid transporters. However, placental nutrient provision remains insufficient to maintain normal fetal growth in the P0 mouse.

It is important to acknowledge that the expression of glutamine and glutamate transport proteins was assessed in membrane‐enriched placental homogenates. This method was chosen because there is currently no established method to isolate the basal membrane of the mouse placenta, and isolation of the apical membrane (Kusinski *et al*. 2010) would require samples to be pooled for sufficient sample size and thus would lose the advantage of paired comparisons. It is possible that changes in protein expression in the apical maternal‐facing plasma membrane are not detected optimally if expression by other membranes in the homogenate makes a substantial contribution to the transport proteins detected.

Taken together, the data presented in this paper indicate that there are different strategies to compensate for small placental size in normally grown (WT, C57BL/6J) and growth‐restricted (P0) fetuses. In WT litters, the lightest placenta employs strategies to maintain appropriate fetal growth, which is supported by the findings that *K*_mf_ of glutamine and glutamate is significantly higher across the lightest *versus* heaviest placentas towards term. Conversely, P0 placentas fail to support a fetus of an appropriate weight by term (57% of fetuses are beneath the 5th centile by E18.5) and the *K*_mf_ of glutamate is similar between P0 and WTL fetuses at E18.5. Total maternofetal delivery of glutamine to the P0 fetus is reduced in P0 *versus* WTL at E18.5, which may also help to explain the reduction in fetal weight observed.

Unlike findings in sheep, pig, rhesus monkey and human (Stegink *et al*. 1975; Pitkin *et al*. 1979; Vaughn *et al*. 1995; Self *et al*. 2004; Day *et al*. 2013), our studies indicate that glutamate is delivered to the fetus, presumably across the BM. Whilst we cannot categorically rule out that this glutamate is metabolised, e.g. into glutamine, glucose or lactate, the time course and previous studies (Stegink *et al*. 1975) suggest that this is unlikely. Radioactive counts in the fetus reflect the amount of radiolabelled glutamate taken up by the placenta (via system X_AG‐_ isoforms). A mechanism of glutamate efflux in the human placenta is via exchange by the organic anion transporter/transporting polypeptides OAT4/OATP2B1. However, OAT4 is not present in the mouse (Rizwan & Burckhardt, 2007; Koepsell, 2013).

In all cases where *K*_mf_ is used to assess amino acid transfer across the placenta in mice, it is possible that the time taken for the radiolabelled isotopes to equilibrate with intracellular metabolic compartments/amino acid pools that exist within the placenta (Velázquez *et al*. 1976; Day *et al*. 2013; Cleal *et al*. 2018) could underestimate the true clearance over the time course of the experiment.

In conclusion, this study provides the first evaluation of maternofetal transfer of glutamine and glutamate *in vivo*, with a specific focus upon the contribution of placental size and sex of the fetus to these transfer processes. We provide evidence of adaptation of placental *K*_mf_ of glutamine and glutamate in relation to placental size in WT mice. Whilst glutamine transfer was also higher for P0 *versus* WTL at both experimental time points, FGR remained evident. The exact mechanisms that mediate these differences have not been fully elucidated and therefore should be the focus of future research. Whilst these studies in mice enable quantification of radiolabelled amino acids in the fetus, future efforts should also assess placental glutamine and glutamate uptake in human FGR if we are to better understand the fundamental pathophysiology of this disorder. Should the data here be translatable to women, they would indicate that placental uptake of glutamine and glutamate, and fetal delivery of glutamine, are compromised in FGR. Alongside glutamine and glutamate placental uptake studies, future studies should also assess umbilical levels of glutamine and glutamate in well‐defined cohorts of human FGR, as opposed to the SGA cohorts previously investigated (Ivorra *et al*. 2012; Alexandre‐Gouabau *et al*. 2013; Sanz‐Cortés *et al*. 2013). These studies will be important moving forward, particularly in the context of the proposition of glutamine/glutamate as a potential biomarker of placental dysfunction in women (Macnaught *et al*. 2015). Ultimately, studies such as this one which help to understand fundamental placental transport processes in health and disease are likely to be important in providing the knowledge to effectively design therapies for placental dysfunction.

## Additional information

### Competing interests

There are no competing interests.

### Author contributions

C. P. Sibley, S. L. Greenwood, M. R. Dilworth and K. R. McIntyre designed research; K. R. McIntyre, C. E. Hayward and M.R. Dilworth performed research; K. R. McIntyre analysed data; K. R. McIntyre, S. L. Greenwood and M. R. Dilworth wrote the paper. All authors approved the final manuscript.

### Funding

The work herein was supported by a Career Development Fellowship Award from the Medical Research Council (MR/K024442/1) awarded to M.R.D. and a Medical Research Council Doctoral Training Partnership PhD studentship (1 512 341) awarded to K.R.M.
